# Automatic detection of facial expressions during the Cyberball paradigm in Borderline Personality Disorder: a pilot study

**DOI:** 10.3389/fpsyt.2024.1354762

**Published:** 2024-05-28

**Authors:** Iván Arango-de-Montis, Adriana Reyes-Soto, Alejandra Rosales-Lagarde, Marta-Lilia Eraña-Díaz, Enrique Vázquez-Mendoza, Andrés Rodríguez-Delgado, Jairo Muñoz-Delgado, Isaac Vázquez-Mendoza, Erika Elizabeth Rodriguez-Torres

**Affiliations:** ^1^ Dirección de Servicios Clínicos, Instituto Nacional de Psiquiatría "Ramón de la Fuente Muñiz", Mexico City, Mexico; ^2^ Dirección Adjunta de Investigación Humanística y Científica, Consejo Nacional de Humanidades, Ciencias y Tecnologías (CONAHCYT), Mexico City, Mexico; ^3^ Facultad de Ciencias Químicas e Ingeniería, Universidad Autónoma del Estado de Morelos, Cuernavaca, Morelos, Mexico; ^4^ Área Académica de Matemáticas y Física, Universidad Autónoma del Estado de Hidalgo, Hidalgo, Mexico; ^5^ Dirección de Investigaciones en Neurociencias, Instituto Nacional de Psiquiatría "Ramón de la Fuente Muñiz", Mexico City, Mexico

**Keywords:** borderline personality disorder, cyberball paradigm, social exclusion, face, emotions, non-verbal expression, pattern analysis, area under the curve

## Abstract

Borderline Personality Disorder (BPD) symptoms include inappropriate control of anger and severe emotional dysregulation after rejection in daily life. Nevertheless, when using the Cyberball paradigm, a tossing game to simulate social exclusion, the seven basic emotions (happiness, sadness, anger, surprise, fear, disgust, and contempt) have not been exhaustively tracked out. It was hypothesized that these patients would show anger, contempt, and disgust during the condition of exclusion versus the condition of inclusion. When facial emotions are automatically detected by Artificial Intelligence, “blending”, -or a mixture of at least two emotions- and “masking”, -or showing happiness while expressing negative emotions- may be most easily traced expecting higher percentages during exclusion rather than inclusion. Therefore, face videos of fourteen patients diagnosed with BPD (26 ± 6 years old), recorded while playing the tossing game, were analyzed by the FaceReader software. The comparison of conditions highlighted an interaction for anger: it increased during inclusion and decreased during exclusion. During exclusion, the masking of surprise; i.e., displaying happiness while feeling surprised, was significantly more expressed. Furthermore, disgust and contempt were inversely correlated with greater difficulties in emotion regulation and symptomatology, respectively. Therefore, the automatic detection of emotional expressions during both conditions could be useful in rendering diagnostic guidelines in clinical scenarios.

## 1 Introduction

Borderline Personality Disorder (BPD) is understood as a persistent pattern of difficulty with emotion regulation, impulse control, identity diffusion, interpersonal conflict, and social cognition impairment. These alterations in the recognition and differentiation of the self and others’ mental states, contribute to affective instability, particularly in social contexts (1). A common feature of BPD is the feeling of abandonment, even though it might not always be present. Patients tend to identify ambiguous pictures as angry faces (2), increasing the frequency at which they experience profound negative emotions, impulsive behaviors, and mistrust in their social interactions.

Nevertheless, the facial expression of patients with BPD during social interactions has been less often studied. Noteworthy, while Staebler et al. (3) evaluated the facial expression of emotions during the Cyberball paradigm, a virtual game to evaluate social interaction and ostracism (4), some issues remained uncovered in such study. In the present work, they were distinctly addressed.

In Staebler et al. (3), two coding systems were employed; one was based on judgment agreement and the second method was an interpretation of the Emotional Facial Action Coding System (EMFACS) under positive, negative, and mixed emotions by a computer program. Instead, a completely automatic analysis was used here. FaceReader (5) is the first commercially available Automated Facial Coding software to assess primary and secondary emotions in facial expressions as well as valence, arousal, head movement, and heart rate; it also renders high resolution and spares the manual coding and judgment agreement needed for the EMFACS implementation.

The term “condition of exclusion” in the Cyberball paradigm does not entail literal and absolute exclusion of the participant. While participants are indeed included in the game throughout the condition of inclusion, under the condition of exclusion, the first interval comprises inclusion, and then, the ostracism properly begins. In particular, Staebler et al. (3) did not evaluate the facial expressions of the complete Cyberball paradigm, as they evaluated them after the tenth ball throw; in consequence, the first period of the condition of exclusion, when the subject is still participating in the game, was not considered.

Williams (4) identified three stages of the time intervals of the Cyberball paradigm: reflexive, reflective, and resignation. The reflexive stage develops while the game is going on. Therefore, herein, analyses of emotional expression intensities throughout the game and its temporal segmentation during the reflexive stage were conducted in female patients with BPD. In Staebler et al. (3), a comparison was made among four different groups, two consisting of patients with BPD and two consisting of healthy controls. Hence, a repeated measure analysis of the non-verbal expression, including both conditions, was not carried out; thus, a complete temporal analysis of the Cyberball paradigm is lacking. Moreover, the joint effects of the conditions of inclusion and exclusion on facial expressions were in here explored using the FaceReader software.

Although patients with BPD were not compared with a control group, their patterns were randomly shuffled to create a synthetic control group for comparison, as in Chichilnisky (6); the methodology is detailed in Section 2.5.2. As stated by Staebler et al. (3), patients with BPD exhibited more blended facial expressions (displaying features of mixed and different emotions) and masking of emotions (covering a negative emotion with smiling) than healthy controls; likewise, even when masking was not statistically significant between conditions, this behavior was remarked during exclusion compared to inclusion. Here, the comparison of masked emotions between conditions was investigated. 

In Staebler et al. (3), the percentage of instances of receiving the ball for the exclusion procedure was 13.3%, while in Gerra et al. (7) it was lessened up to 10%. Here, the protocol was designed to ostracize the subject with greater severity; therefore, the individual received the ball at a percentage of 6.6%.

Consequently, the following hypotheses were tested:

- Clinical measurements are correlated within themselves, i.e., symptomatology and dysregulation are positively correlated.
- Subjective ratings of the Need Threat Scale (NTS) are higher after the condition of exclusion versus inclusion.
- The intensity of negative emotions (anger, disgust, contempt, fear, and sadness) expressed during the condition of exclusion is higher compared to the condition of inclusion.
- The distributions of the patterns of emotions under inclusion and exclusion are different compared to a shuffled and randomized pattern.
- The second and third segments of the condition of exclusion versus the respective ones under the condition of inclusion trigger more negative emotions (anger, disgust, contempt, fear, and sadness).
- We expected blended and masked emotions to be different during the condition of exclusion versus the condition of inclusion.
- Some intensities of emotions, like anger and contempt, are positively correlated with the abovementioned scales, looking forward to finding useful markers of emotional dysregulation and symptomatology.

## 2 Method

### 2.1 Subjects

The Ethics Committee of the National Institute of Psychiatry “Ramón de la Fuente Muñiz”˜ (INPRFM) approved the project. Patients agreed to participate in the study by signing informed consent. The sample comprised 14 women with a confirmed diagnosis of BPD according to the DSM-IV diagnostic criteria, who regularly attended the Borderline Personality Disorder Clinic of the INPRFM. BPD patients were recruited in outpatient settings. BPD onset can appear in adolescence or early adulthood and the average age of the recruited patients was 26 ± 6 years old, ranging from 19 to 35 years old. They had an average schooling of 12.7 ± 4 years. Socio-demographic and clinical data is presented in **Table 1**.

**Table 1:** Description of patients’ socio-demographic and clinical characteristics.

Patient socio-demographic characteristics	
Age, mean (SD)	26 (6)
Education (years), mean (SD)	12.79 (4)
Students %	21.43%
Housekeepers %	57.14%
Employed %	14.29%
Unemployed %	7.14%
Singles %	71.43%
Widows %	14.29%
Married %	14.29%
Mothers %	28.57%
Childless women %	71.43%
Clinical characteristics	
SCID-II, mean (SD)	12.36 (2)
BIS-15, mean (SD)	49.79 (13)
Cognitive impulsiveness, mean (SD)	15.93 (5)
Motor impulsiveness, mean (SD)	13.79 (4)
No planning impulsiveness, mean (SD)	19.93 (5)
BEST, mean (SD)	44.00 (11)
BEST A, mean (SD)	30.00 (6)
BEST B, mean (SD)	13.43 (4)
BEST C, mean (SD)	12.07 (3)
DERS-E, mean (SD)	81.08 (15)

Abbreviations correspond to: Structured Clinical Interview for DSM-IV (SCID-II), Barratt Impulsiveness Scale (BIS-15), Borderline Evaluation of Severity Over Time (BEST), and Difficulties in Emotion Regulation Scale, Spanish version (DERS-E).

All of them were diagnosed with BPD, as stated above, and had received four sessions of a psychoeducational treatment. Only 3 out of the 14 women had received Dialectical Behavioral Therapy (DBT). Patients did not present any neurological disease or manic or psychotic episodes that would jeopardize their performance in this study. Patients were not agitated, aggressive, or suicidal during the study. None of the patients decided to quit without completing the evaluations. For all patients, except for one, Major Depressive Disorder (MDD) was the most frequent comorbidity of Axis 1; for the one excepted patient, Obsessive Compulsive Disorder was the respective comorbidity. In 9 out of the 14 patients, MDD was recurrent, and yet 13 out of the 14 subjects were in remission. One patient was relapsing, and in two of them, it was persistent.

Patients were prescribed a total of 33 medications. Of these, 48.48% (16/33) were antidepressants (Fluoxetine, Sertraline, Duloxetine, Venlafaxine, Fluvoxamine, Mirtazapine, and Citalopram), 24.24% (8/33) were antipsychotics (Quetiapine and Risperidone), 21.42% (7/33) were anticonvulsants (Magnesium Valproate, Topiramate, and Pregabalin), and 6.06% (2/33) were benzodiazepines (Clonazepam). All fourteen subjects took at least one antidepressant, 7 of them were also prescribed at least one antipsychotic, 5 were taking anticonvulsants, and 2 reported the consumption of at least one benzodiazepine.

### 2.2 Clinimetric evaluation

The subjects were evaluated individually using the following tests:

#### 2.2.1 Structured Clinical Interview for DSM-IV Axis II Personality Questionnaire (SCID-II)

The SCID-II (8) is a semi-structured interview consisting of 119 yes/no questions. It can be used to formulate Axis II diagnoses, both categorical and dimensional. The psychometric properties of the SCID-II have been widely studied. According to a review of the Encyclopedia of Clinical Neuropsychology, Gorgens (9), SCID-II internal consistency coefficients ranged from 0.71 to 0.94 and inter-rater reliability coefficients ranged from 0.48 to 0.98 for categorical diagnosis. Also, inter-rater reliability on dimensional judgments ranged from 0.90 to 0.98. The validity of the SCID-II has been guided by the “LEAD standard”, based on longitudinal studies performed by experts. The diagnostic power of the SCID-II was 0.85 or greater for five personality disorders. Specifically, questions number 90 to 105 of the SCID-II assess symptoms for BPD (10).

#### 2.2.2 Barratt Impulsiveness Scale (BIS-15)

BIS-15 is the currently widely used version of the Barratt Impulsivity Scale. It has been psychometrically validated in Spanish adults and adolescents (11). Internal consistency was 0.793 and test-retest reliability was 0.80. A three-factor structure was confirmed by factor analysis, accounting for 47.87% of the total variance in BIS-15 total scores. This is a self-report scale measuring three areas of impulsiveness: 1) attentional, 2) motor and 3) non-planning impulsiveness. This Spanish version consists in 15 items scored from 0 to 4.

#### 2.2.3 Borderline Evaluation of Severity Over Time (BEST) (Version 1.7)

BEST is composed of 15 self-report items measuring the severity of the main symptoms of patients with BPD, e.g., mood reactivity, identity alteration, unstable relationships, paranoia, emptiness, suicidal thinking, and negative actions, on a five-point Likert scale (12). Cronbach’s *α* coefficients for patients with BPD and controls were respectively, 0.86 and 0.90. Test-retest reliability was moderate (*r* = 0.62, *n* = 130, *p* ≤ 0.001) (12).

#### 2.2.4 Need Threat Scale

This scale consists of 14 items that evaluate the intensity of the person’s adverse experience during the game (13). The total score is 140, where higher score indicate more ostracism. The NTS was administered after each condition; i.e., inclusion and exclusion, as the most suitable method to measure subjective perception after the Cyberball paradigm administration. Convergent and discriminant validity studies showed subscales of the NTS and most of the ten Sheldon subscales (the Sheldon scale measures autonomy, relatedness, competence, self-esteem, popularity-influence, physical thriving, self-actualization-meaning, money-luxury security and pleasure-stimulation) were correlated (*α* = 0.71 to 0.79); factor analyses found the four-factor structure of the NTS was not supported and thus the four needs seemed to be overlapped rather than distinct (14).

#### 2.2.5 Difficulties in Emotion Regulation Scale, Spanish version, DERS-E

This scale comprises 24 items that evaluate the capacity of being dysregulated in four domains:

Nonacceptance of Emotional Responses, Difficulties Engaging in Goal-Directed, Lack of Emotional Awareness, and Lack of Emotional Clarity. The subscales of Cronbach´s α range from 0.85 to 0.68 according to Hervás and Jódar (15). The validity through contrasted groups and the correlation with concurrent scales showed significant results (Pearson’s *r* coefficient ranging from 0.51 to 0.76, *p<* 0.05).

### 2.3 Materials

#### 2.3.1 The Cyberball paradigm

Version 5 of the Cyberball sotware was used in the study, Previous versions of the software can ran of PC and/or Mac platforms while this version provides an online executable alternative. The participants were scheduled to be online, playing with two virtual players. The data from the game, including the total number of players’ ball throws, and the number of mouse clicks, were collected for analysis by the Cyberball software itself (the information on the number of clicks was not considered for this study). The game consisted of throwing a ball with two additional players. However, the participants were interacting with programmed virtual players, despite their initial perception of playing with real people.

In the present study, each condition of the Cyberball paradigm was divided into three temporal segments. Such segmentation was implemented to discriminate and consider the moments when participants were effectively excluded, specifically under the condition of exclusion. The average duration of the Cyberball paradigm for the current participants was 2 minutes and 25 seconds. However, the initial 14 seconds were disregarded given that time was used by the experiment monitor to type the names of the participants and select the respective experimental condition in the game. Therefore, the recordings displayed two minutes and 9 seconds of relevant material which was segmented as follows:

- segment 1: from 0:00 to 0:59 minutes, during which the patient’s facial expressions under the respective experimental condition are displayed, 45 seconds; i.e., from 0:14 to 0:59 minutes;
- segment 2: from 1:00 - 1:45 minutes, during which the patient’s facial expressions under the respective experimental condition are displayed, 45 seconds;
- segment 3: from 1:46 - 2:25 minutes, during which the patient’s facial expressions under the respective experimental condition are displayed, 39 seconds.

During the condition of inclusion, a total of 30 ball throws were made. One third of the ball throws were programmed to be received by the patient. In the first, second, and third segments, the ball was thrown to be caught by the participant a total of four, four, and two times, respectively. Regarding the condition of exclusion, the number of 30 ball throws remained unchanged; notwithstanding, only two of them were programmed to be received by the participant during this part of the experiment. The occasions in which the participant received and threw the ball occurred in the first segment exclusively.

Each participant could decide whom to throw the ball to among the other two players and when to do it. The NTS was conducted after the culmination of each experimental condition. As previously mentioned, in the Cyberball paradigm, the participant must throw the ball toward one of the two other players, this is emphasized since leads to time variations in the final duration of the game and, consequently, affects the duration of the video recordings, which ranges from 2 minutes and 5 seconds to 2 minutes and 32 seconds.

#### 2.3.2 FaceReader 7.0

The software first detects a person’s face based on the Viola and Jones algorithm. Subsequently, two combined methods are applied. The first method, Face modeling and classification, is based on an Active Appearance Model (AAM) which, detects over 500 key points of the face and refers to previous databases to estimate the variations of the new face image sampled compared to an average face (5). The second method, Deep face classification, uses a deep artificial intelligence algorithm of pattern recognition to identify facial expressions according to (5). Afterwards, the Principal Component Analysis (PCA) is applied to reduce the model dimensions. With the gathered information, a neural network is trained to classify the emotions.

To train the neural network, 10000 manually rated images were considered. The Deep face classification method uses deep face classification from image pixels and can be used alternatively when the Face modeling and classification method provides no informative results. These models calculated the probability and intensity scores for facial expressions on a continuous scale from 0 to 1 (5). FaceReader accurately identifies facial expressions, with a reported agreement between manual and automatic detection ranging from 89.6% for scared expressions to 99% for happy expressions (5).

In the absence of a neutral face captured prior to the experimental conditions, a continuous calibration was performed. This procedure allows the adaptation to the particular face bias. According to the FaceReader’s manual (5), this software continuously averages the facial expression intensities, correcting them. Therefore, the intensity of the current frame considers the average intensity of previous frames as follows (Equation 1):


(1) $$\text{Expression intensity }=\text{ max}\left(0,\frac{l_{a}-l_{m}}{1-l_{a}}\right),$$

where $l_{a}$ is the expression intensity in the current frame and $l_{m}$ is the average expression intensity over all frames before the current frame. If $\frac{l_{a}-l_{m}}{1-l_{a}}<0$ , the previous equation is equal to 0; i.e., the expression intensity is also zero.

Additionally, the intensity of neutral is given by (Equation 2):


(2) $$\text{Intensity Neutral }=\frac{N_{a}+(1-l_{\max\;m})}{2},$$

where $N_{a}$ is the intensity of Neutral classified by FaceReader in the current frame and $l_{\max\;m}$ denotes the maximum average intensity of all emotions in all the frames before the current one.

The FaceReader software classifies facial expressions into seven categories: happiness, sadness, anger, surprise, fear, disgust, and contempt; i.e., the primary emotions.

### 2.4 Procedure

The patients completed the clinimetric evaluations several days before the study began. If they met the inclusion criteria, they were invited to participate in this investigation. Under both experimental conditions, the patients with BPD were carefully video recorded with an SJCAM HD 1080P camera, which was located in a special room at the INPRFM illuminated with a white light bulb.

**Figure 1:**
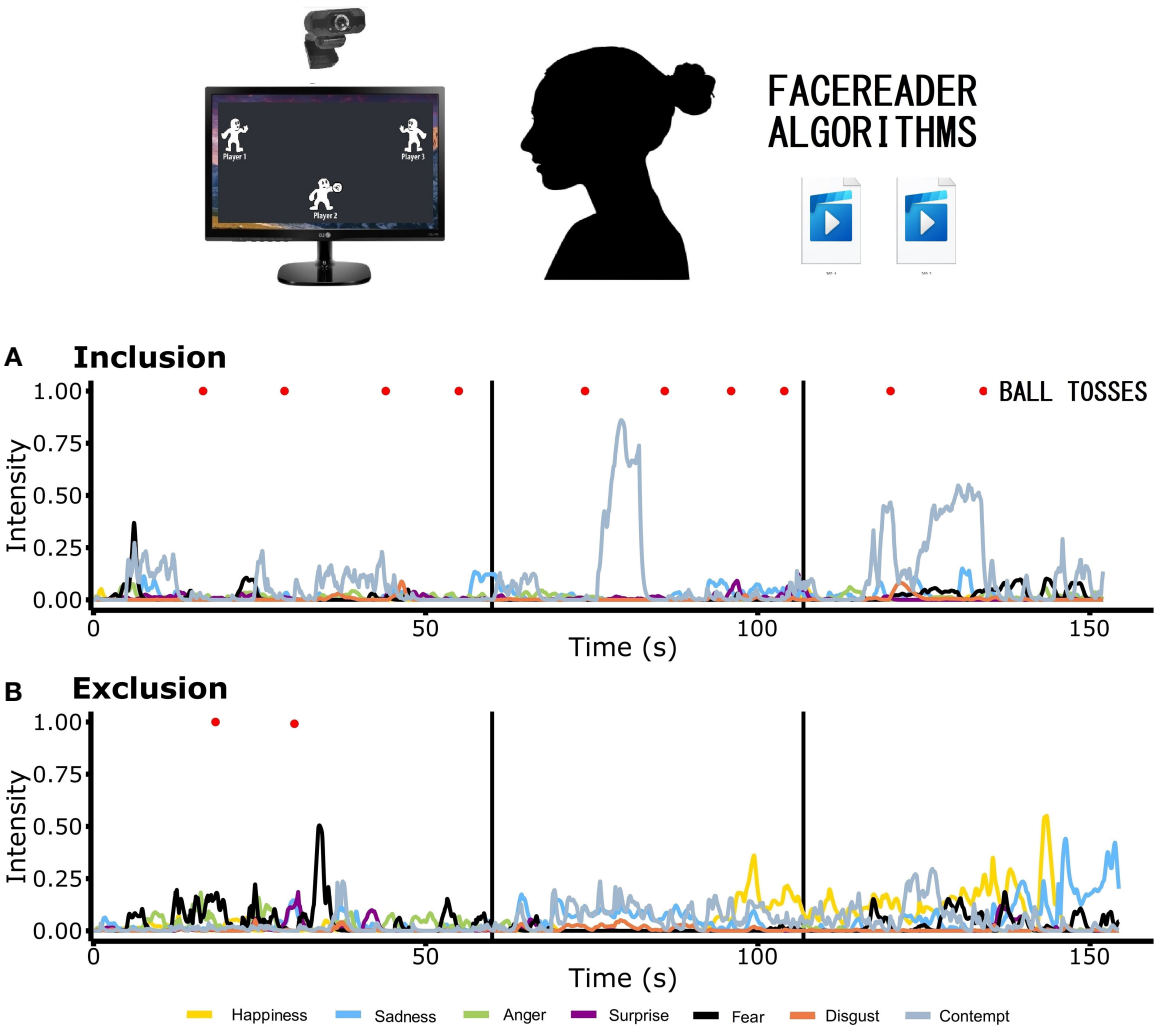
Experimental protocol. Participants were video recorded while playing the Cyberball paradigm under each condition of the game, i.e., inclusion and exclusion. Experimental conditions were video recorded separately. The videos were subjected to the FaceReader algorithms. The values of primary emotions of one patient with Borderline Personality Disorder are displayed in the two graphics, corresponding to **(A)** inclusion and **(B)** exclusion, presenting the temporal segmentation. Two main analyses (not shown) were conducted: 1a) second-by-second pattern analyses (percentage of subjects displaying the highest intensity of a particular emotion in that second) for each condition; 1b) pattern analysis of the sum of the percentages of 15 seconds and, the difference of these percentages between conditions (exclusion minus inclusion). 2) Adjusted area under the curve: 2a) individual and 2b) group analyses.

Standard instructions were given about the need to visualize the scenario in which the other players would be present, etc. (13). Subjects were seated 50 cm away from a 14-inch screen. When participants had the chance to throw the ball, they could choose either player 1 (on the left) or player 3 (on the right) by clicking on them with the mouse. After instructing the patients, the experiment monitor started to record the video and went out of the room. Once the patient was in private, the game began and the recording was stopped until the end of the game. The general experimental procedure is illustrated in **Figure 1**.

### 2.5 Statistical analysis

#### 2.5.1 Socio-demographic data and clinimetric assessments

Data from the scales and the collected patterns were analyzed using Excel and the Statistical Package for the Social Sciences (SPSS) v.17. Frequency and percentages were calculated for the categorical variables, while means and standard deviations were computed for the scales. Spearman’s correlations were conducted between scores of scales (BEST and DERS-E, BEST and BIS-15 and BIS-15 and DERS-E). Afterwards, these scales were correlated with the AUC of each emotion. The reported values from the variables recorded by the FaceReader software were analyzed using the following two methods:

#### 2.5.2 Pattern analysis: second-by-second pattern and temporal segmentation analyses

Given that emotions can be considered as emotional states if they last at least 0.5 seconds (16), a second-by-second descriptive analysis was carried out with the original outputs of the signals, as shown below. The software can be set up to store and process 1, 15, 30, or 60 frames per second. For this study, the FaceReader software was set to default mode, thus the frame rate was automatically determined by the program configurations (16). Therefore, 30 frames per second were recorded for 12 of the subjects, while 60 frames per second were captured for the remaining two patients. Considering the complete videos recorded from the 14 participants’ faces, the total number of frames per second ranged from 5,362 to 12,878 for both conditions.

For each emotion, we averaged the 30 or 60 scores reported, depending on the frame rate used, to obtain a single value representing the emotion score per second. After calculating the emotion score per second of each emotion, the patient’s most intense emotion for each second was defined as the emotion with the highest score in the respective second. The above procedure was applied to the outputs of the video recordings of the faces of all 14 participants under each experimental condition. Afterwards, for each condition, the percentage of patients displaying each primary emotion was determined second by second. To streamline the analysis, we averaged the percentage of patients’ emotions every 15 seconds (except the last interval, which consisted only of 10 seconds), dividing the first segment into four intervals, while the second and third segments were divided into three intervals each.

In each interval, the most and least common emotion was found. Finally, it was determined which emotion showed the greatest increase and the greatest decrease in each patient due to the social exclusion for each interval. Therefore, for each emotion considered, the difference between the percentage of patients who showed the respective emotion during exclusion and the one during inclusion was obtained.

As previously stated, we aim to investigate whether the distributions of the patterns of emotions under inclusion and exclusion are different compared to a shuffled and randomized pattern. Consequently, the reported frequencies under each experimental condition were shuffled second by second, obtaining a white noise version of them to perform the comparisons.

Shuffling analyses create new patterns by randomizing the data sets. The patterns of both conditions were tested against their respective random-shuffled data sets created by randomly shuffling the frequencies of the original data sets, as shown in **Algorithm 1**. White noise is inherently random; furthermore, each white noise sample is statistically independent of the others, and there is no correlation between successive samples. This randomness property makes white noise useful in various applications, such as modeling stochastic processes, simulating random events, and providing a baseline for comparison in statistical analyses (6, 17–20).

When used in data analysis or modeling, white noise is sometimes assumed as a null hypothesis; i.e., a synthetic control group where the patterns observed in the data are due to random variation inherent in the data *per se*, rather than any underlying factors, such as a dynamical system attractor. Deviations from white noise behavior in a signal might indicate the presence of underlying patterns, trends, or systematic effects that warrant further investigation. Additionally, techniques such as spectral analysis can be employed to examine the frequency content of a signal and assess its deviation from the characteristics of white noise (18–20)).

Algorithm 1. Shuffled matrix for the comparison of the patterns of both conditions 
1: Define *dfsorted* as the matrix for shuffling the data of video 1
2: Initialize a matrix called *Add_random_V IDEO*1 with 346 rows and 7 columns
3: **for** *i* from 1 to 173 **do**
*⊳* Adjust according to the number of rows
4: *Add_random_V IDEO*1[*i* ∗ 2 − 1][1: 7] ← row *i* of *dfsorted  ⊳* Odd rows are video 1 data
5:  *Add_random_V IDEO*1[*i* ∗ 2][1: 7] ← Random permutation of numbers from 1 to 7 *⊳*Even rows have random numbers for shuffling
6: **end for**

7: Obtain video 1
8: **for** *i* from 1 to 173 **do**  *⊳* Adjust according to the number of elements to be sorted
9:  *order* ← row *i* ∗ 2 of matrix *Add_random_V IDEO*1  *⊳*Only even rows have been rearranged according to the shuffling or random numbers
10:  *B, IX* ← Sort the elements of *order*
11:  *sortedV ideo*1[*i*][1: 7] ← Row *i* ∗ 2 − 1 of *Add_random_V IDEO*1 sorted according to indices *IX*
12: **end for**

#### 2.5.3 Area under the curve (AUC) analysis

A subject-per-subject standardized analysis with the seven emotions was carried out, and the AUC was obtained. To determine the degree of masking, the AUC respective to happiness was summed to the AUC of each emotion, independently. Group differences were sought between and within conditions and time intervals for every emotion. For the implementation of the AUC analysis, the reported FaceReader outputs were processed according to the following procedure:

1. Plotting facial expression profile recorded during the conditions of inclusion and exclusion.
2. Determining the total number of frames from the video recorded during each experimental condition, exclusively while the game was running; i.e., disregarding the video’s initial part when patients typed their names and chose the respective experimental condition. As previously mentioned, each video had a variable duration. Therefore, the total number of frames from each video is the product of the video length in seconds and the frame rate at which the video was recorded, 30 frames per second (*n* = 12 participants) or 60 frames per second (*n* = 2 participants).
3. Calculating the Area Under the Curve (AUC) according to the trapezium method (21) for each emotion and during the respective experimental conditions.
4. Standardizing the time interval used to compute the AUCs by multiplying the emotion intensity score per second and the number of frames.

The above protocol was implemented to every participant’s data. Additionally, differences between the emotions expressed during both experimental conditions, with no segmentation, were sought by performing a one-way repeated measures ANOVA and Wilcoxon tests. The AUC for every emotion expressed during each of the three segments was computed, and a two-way repeated-measures ANOVA was performed to assess differences between conditions per segment. Afterwards, pairwise comparisons (paired Student’s t-test) with Bonferroni corrections were conducted, to explore differences between segments.

Data normality was verified by conducting Shapiro-Wilk tests and Q-Q plots, as implemented in *rstatix* and *ggpubr* R packages. In cases the normality assumption was not met, data normalization was performed using the *bestNormalize* R package to determine the optimal transformation for each data set. Normalizations were carried out according to hyperbolic arcsine transformation for anger, happiness, sadness, and surprise; ordered quantile normalizing transformation for fear; and Yeo-Johnson transformation for disgust. Outliers were identified using the *identify_outliers* function from the *rstatix* R package. Outliers are defined as data points that fall above the *Q*_3 +_ 3*IQR* or below the *Q*_1_ − 3*IQR* thresholds, where *Q*_1_ and *Q*_3_ denote the first and third quartile and, *IQR* represents the interquartile range. For these analyses, ANOVAs were performed with and without extreme outliers, with and without the Benjamini-Hochberg corrections, respectively. In general, both analyses yielded the same results with and without outliers. Therefore, the herein-reported results include outliers.

All the analyses presented in this section were performed using R (version 4.1.0 – “Camp Pontanezen”) R Core Team (22). Given that multiple ANOVAs were performed, the *p*-values were corrected using the Benjamini-Hochberg method. This correction was implemented using the *p.adjust* function, from the *stats* R package, to control the false discovery rate (FDR).

## 3 Results

### 3.1 Correlations between scales

In **Table 2**, Spearman’s correlations between clinimetric scales are reported. The SCID-II scores are significantly correlated with the BIS-15 (*r* = 0.37*, p<* 0.05), BEST (*r* = 0.65*, p<* 0.01), and DERS-E (*r* = 0.68*, p<* 0.01) scales. Additionally, the BIS-15 scale is significantly correlated with the DERS-E (*r* = 0.23*, p<* 0.05) scale. Similarly, the scales BEST and DERS-E were positively correlated (*r* = 0.32*, p<* 0.05).

**Table 2:** Spearman’s correlations between the clinical scales, with the Bonferroni correction for the *p*-value.

	SCID-II	BIS-15	BEST	DERS-E	NTS
**SCID-II**	1	**0**.37*	**0**.65**	**0**.68**	0.14
**BIS-15**	**0**.37*	1	0.16	**0**.23*	−0.02
**BEST**	**0**.65**	0.16	1	**0**.32*	−0.08
**DERS-E**	**0**.68**	**0**.23*	**0**.32*	1	0.12
**NTS**	0.14	−0.02	−0.08	0.12	1

SCID-II vs. BIS-15 (p = 0.018), SCID-II vs. BEST (p = 0.001), SCID-II vs. DERS-E (p = 0.0001), SCID-II vs. NTS (p = 0.062) BIS-15 vs. BEST (p = 0.057), BIS-15 vs. DERS-E (p = 0.044), BIS-15 vs. NTS (p = 0.094), BEST vs. DERS-E (p = 0.027), BEST vs. NTS (p = 0.076), and DERS-E vs. NTS (p = 0.069). The respective symbology has been reported (*, p< 0.05; **, p< 0.01).

### 3.2 Need threat scale

The mean and standard deviation scored on the NTS for the condition of inclusion were 78.50 ± 29, while the corresponding scores on the NTS for the condition of exclusion were 110.57 ± 29. The statistical difference between them was significant (*t*_13_ = 5.25*, p<* 0.0001).

### 3.3 Cyberball

There were no significant differences between the duration, in seconds, of both experimental conditions (condition of inclusion = 129.92 ± 11; condition of exclusion = 128.40 ± 2, *t*_3_ = 0.12*, p<* 0.90).

### 3.4 Second-by-second analysis: frequencies of emotions according to their highest mean intensity

In **Table 3**, the frequencies converted to percentages of the pattern analysis are shown. Regarding the condition of inclusion, in the first segment (seconds 1 to 59): sadness was the most common emotion displayed during the first (seconds 1 to 15), second (seconds 16 to 30), and fourth (seconds 45 to 59) intervals, whereas contempt was the most common during the third (seconds 31 to 44) interval. Concerning the condition of exclusion, in the first segment, sadness was also the most common emotion only during the first (seconds 1 to 15) and fourth interval (seconds 45 to 59), in contrast to the condition of inclusion. Likewise, contempt was also the most common during the second (seconds 16 to 30) and third intervals (seconds 31 to 44). During the second segment (seconds 60 to 106) of the condition of inclusion, sadness was the most predominant emotion while fear was the least expressed emotion during the entire segment. Respecting the second segment (seconds 60 to 106) of the condition of exclusion, sadness was the most common emotion displayed during the second (seconds 76 to 90) and third (seconds 91 to 106) intervals; however, during the first interval (seconds 60 to 75), contempt was the most common emotion. Regarding the third segment (seconds 106 to 145) of the condition of inclusion, sadness was reported as the most common emotion displayed during the first (seconds 106 to 120) and third (seconds 136 to 145) intervals; similarly, during the second interval (seconds 121 to 135), anger was reported as the most expressed emotion. In the condition of exclusion, third segment (seconds 106 to 145), sadness was the most common emotion during the second (seconds 121 to 135) and third (seconds 136 to 145) intervals, while happiness was the most common during the first interval (seconds 106 to 120). The increases in fear, surprise, and happiness, as well as the decreases in sadness and anger at the end of the condition of exclusion, contrasted to that of inclusion one, were evident. Additionally, sadness held the highest value throughout the entire game.

**Table 3:** Summary of the pattern of emotions.

Segment		1				2			3		
Interval		1	2	3	4	1	2	3	1	2	3
Emotion	Condition										
**Happiness**	**Inclusion**	15.64	24.10	10.99	12.20	15.79	5.10	11.04	10.99	12.48	8.40
	**Exclusion**	12.77	14.95	15.24	20.00	13.44	14.07	21.43	27.25	22.60	32.70
**Sadness**	**Inclusion**	25.68	24.62	29.71	29.89	32.32	32.78	36.70	31.36	26.97	32.50
	**Exclusion**	26.77	20.26	21.90	30.00	25.79	34.54	30.80	23.92	27.29	36.79
**Anger**	**Inclusion**	22.78	21.76	13.92	19.49	14.84	18.69	18.36	23.61	30.77	27.05
	**Exclusion**	24.15	19.96	15.71	16.19	13.41	8.86	14.29	13.37	6.70	7.11
**Surprise**	**Inclusion**	10.26	9.19	9.16	9.41	11.00	9.65	10.86	8.26	4.40	3.76
	**Exclusion**	5.83	15.49	15.24	12.86	13.88	21.21	8.04	13.44	19.78	20.44
**Fear**	**Inclusion**	4.36	5.79	1.90	2.93	1.50	1.72	1.96	0.51	0.56	7.33
	**Exclusion**	4.84	2.89	6.67	4.29	3.85	2.86	3.57	3.37	5.82	0.71
**Disgust**	**Inclusion**	1.94	0.51	0.51	0.51	1.58	2.83	2.88	6.62	7.05	2.58
	**Exclusion**	2.38	1.47	0.48	2.86	1.90	1.90	3.57	1.47	0.00	0.00
**Contempt**	**Inclusion**	19.34	14.03	33.81	25.57	22.97	29.24	18.19	18.66	17.78	18.39
	**Exclusion**	23.27	24.98	24.76	13.81	27.73	16.56	18.30	17.18	17.80	2.25

Frequency, in percentage (%), of patients with Borderline Personality Disorder who displayed each emotion (happiness, sadness, anger, surprise, fear, disgust, and contempt) as the dominant (i.e., the emotion with the highest score) during a Cyberball session, both during social inclusion and exclusion. Intervals consist in: **1**, 1-15; **2**, 16-30; 3, 31-44; **4**, 45-59; **5**, 60-75; **6**, 76-90; 7, 91-105; **8**, 106-120; **9**: 121-135; **10**, 136-145 seconds. Each interval is the average of 15-second percentages. The total percentage for each condition in each column is 100% when the percentages are summed vertically. The data was originally obtained from the FaceReader outputs.

**Figure 2:**
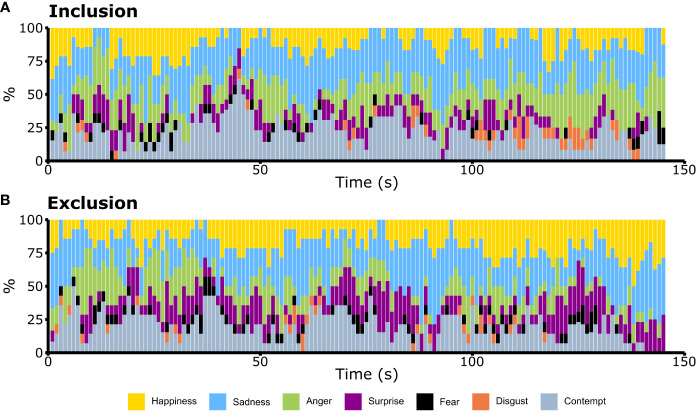
Pattern of emotions in the Cyberball paradigm during **(A)** the condition of inclusion and **(B)** during the condition of exclusion across time. Happiness (yellow), sadness (blue), anger (green), surprise (purple), fear (black), disgust (orange) and contempt (gray). Each bar represents 1 second. For each emotion, it shows the percentage of patients with Borderline Personality Disorder who displayed such emotion as the one scoring the highest value in that second. Sadness (blue) was constantly expressed. Anger (green) increased throughout the condition of inclusion and it decreased in the condition of exclusion. The prominence of happiness (yellow) and surprise (purple) suggested a higher degree of patients showing those emotions during ostracism.

In **Table 4**, the differences in percentages of the reported values under the condition of exclusion minus the ones under the condition of inclusion, per emotion, are presented for each segment and interval. Differences, in absolute value, were greater than ten percentage points; i.e., all differences are greater than 10 or less than -10. In the first segment, contempt attained the greatest increase, whereas surprise and happiness held the greatest increase during the last two intervals. Conversely, anger and contempt reported the greatest decreases, with anger showing greater consistency than contempt. In **Figure 2**, the percentages for the group, per emotion, during the entire game can be visualized.

**Table 4:** Pattern analysis.

Segment	Happiness	Sadness	Anger	Surprise	Fear	Disgust	Contempt
1	-2.88	1.09	1.37	-4.43	0.48	0.44	3.93
1	-9.16	-4.36	-1.79	6.30	-2.89	0.95	**10.95**
1	4.25	-7.80	1.79	6.08	4.76	-0.04	-9.05
1	7.80	0.11	-3.30	3.44	1.36	2.34	***-11.76***
2	-2.34	-6.53	-1.43	2.89	2.34	0.32	4.76
2	8.96	1.76	-9.82	**11.56**	1.14	-0.92	***-12.69***
2	**10.39**	-5.89	-4.08	-2.83	1.61	0.69	0.11
3	**16.26**	-7.44	***-10.24***	5.19	2.86	-5.16	-1.48
3	**10.12**	0.32	***-24.07***	**15.38**	5.27	-7.05	0.02
3	**24.29**	4.29	***-19.94***	**16.68**	-6.61	-2.58	***-16.14***

Differences in percentage values between the conditions of the Cyberball paradigm (exclusion versus inclusion) for each temporal segment, as recorded by the FaceReader software. In bold text, the values that increased; in italic-bold, the values that decreased, in each temporal segment. The increase in happiness and surprise, especially in the last temporal segments, is evident, while contempt initially increased and then decreased. Each condition of inclusion and exclusion in the Cyberball paradigm was divided into three segments (first: 1-59, second 60-106, and third 107-145 seconds). Furthermore, each temporal segment was divided into 4, 3, and 3 intervals. Intervals consist in: **1**, 1-15; **2**, 16-30; **3**, 31-44; **4**, 45-59; **5**, 60-75; **6**, 76-90; **7**, 91-105; **8**, 106-120; **9**: 121-135; **10**, 136-145 seconds.

**Figure 3:**
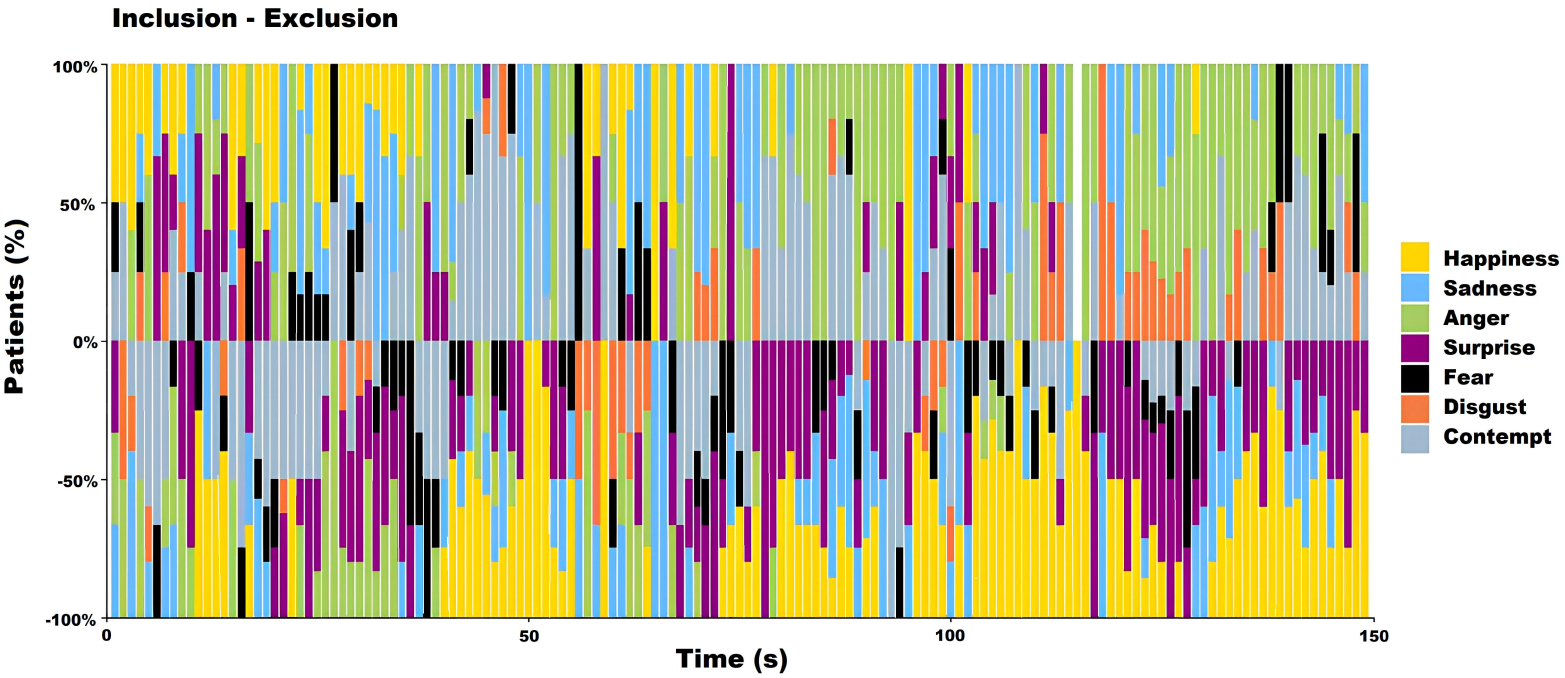
The second-to-second differences between the pattern analyses of the inclusion minus exclusion conditions among participants with Borderline Personality Disorder during the Cyberball paradigm were examined, with frequencies transformed to percentages. An increase in the percentage of participants expressing anger (green) was particularly notable, especially toward the end of the inclusion condition (top). Conversely, in the exclusion condition (bottom), surprise (purple) and happiness (yellow) were more predominantly expressed.

This can be more easily visualized in **Figure 3**, where the differences between the frequencies of the condition of inclusion minus the ones of exclusion were calculated.

The comparative error was remarked due to sample sizes. The small sample sizes were crucial, and even the greatest change of the third interval of happiness, which raised from 8.40% in inclusion to 32.70% in exclusion, lacked statistical significance (comparative error = 28.54, percentage difference = 24.30). Under the assumption of 20 hypothetical subjects, the observed difference became statistically significant (comparative error = 23.88, percentage difference = 24.30).

**Figure 4:**
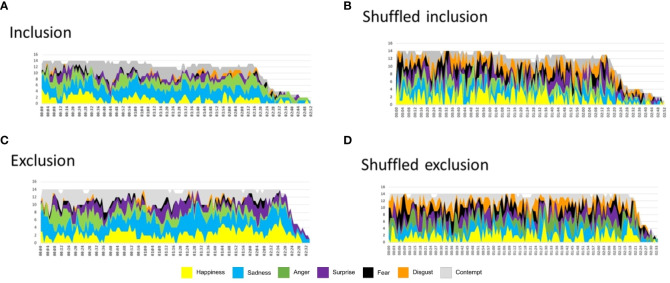
**(A)**. Inclusion Patterns of the primary emotions 1) Happiness, 2) Sadness, 3) Anger, 4) Surprise, 5) Fear, 6) Disgust, and 7) Contempt. **(B)**. Shuffled Inclusion shows that the patterns in A are lost. 1)Happiness vs. Shuffled Happiness (*t* = 2.41*, p<* 0.01); 2) Sadness vs. Shuffled Sadness (*t* = 10.55*, p<* 2.27611*E*−20); 3) Anger vs. Shuffled Anger (*t* = 5.54*, p<* 1.07248*E*−07); 4) Surprise vs. Shuffled Surprise (*t* = 4.41*, p<* 1.78844*E* − 05); 5) Fear vs. Shuffled Fear (*t* = 9.66*, p<* 6.45638*E* − 18); 6) Disgust vs. Shuffled Disgust (*t* = 9.89*, p<* 1.55306*E* − 18); and 7) Contempt vs. Shuffled Contempt (*t* = 5.79*, p<* 3.16769*E* − 08). More details in the text. **(C)**. same as A for exclusion patterns **(D)**. Shuffled Exclusion shows that the patterns in A are lost. 1)Happiness vs. Shuffled Happiness (*t* = 3.76*, p<* 0.0002), 2) Sadness vs. Shuffled Sadness (*t* = 9.34*, p<* 1.04266*E* − 16), 3) Anger vs. Shuffled Anger (*t* = 1.5912*, p<* 0.11), 4) Surprise vs. Shuffled Surprise (*t* = 1.07*, p<* 0.28), 5) Fear vs. Shuffled Fear (*t* = 9.38*, p<* 8.28122*E* − 17), 6) Disgust vs. Shuffled Disgust (*t* = 10.91*, p<* 7.29614*E* − 21), and 7) Contempt vs. Shuffled Contempt (*t* = 4.06*, p<* 7.67073*E* − 05). More details in the text.

In **Figure 4**, the distributions of the shuffled frequencies are represented. Almost all the distributions per emotion and condition and their randomized patterns were statistically significant.

### 3.5 Area under the curve

#### 3.5.1 Individual level

**Figure 5:**
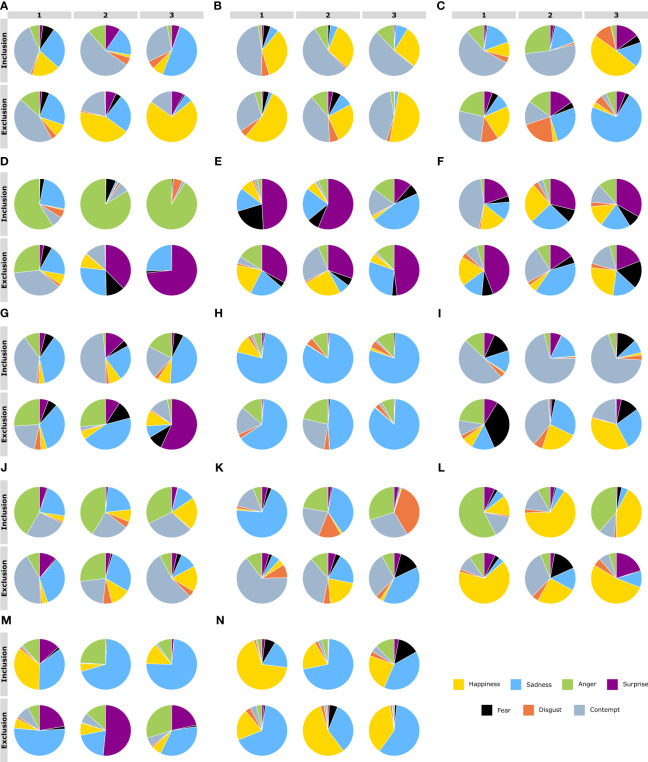
Pie charts displaying the proportions of the area under the curve (AUC) for the seven emotions. The AUC were obtained after the facial emotions of patients with Borderline Personality Disorder were detected by the FaceReader software during the Cyberball paradigm session. Each participant is represented by a capital letter of the alphabet (from letter **A–N**). The proportions of the AUC were obtained for the three time segments and the two conditions. The conditions of inclusion and exclusion were analyzed separately in three AUCs: 1 minute for the first segment (left), and 45 seconds each for the second and third segments (middle and right, respectively). The expression of facial emotions of patients are depicted for social inclusion (above) and social exclusion (below). Each color represents one emotion: yellow is happiness; blue is sadness; anger is green; surprise is purple; fear is black; disgust is orange; and gray is contempt.

As shown in **Figure 5**, different proportions of emotions were found for each of the three segments in every condition, noting several cases of blending and masking. Regarding participants 5D and 5L, anger was predominantly present, while contempt was found in high percentages among several subjects (5A, 5B, 5C, 5F, 5G, and 5I), in the condition of inclusion. Sadness was expressed in many subjects in both conditions (5G, 5H, 5K, 5M, 5N). Some participants (5B, 5F,5L, 5M, 5N) expressed high levels of happiness in both conditions, inclusion and exclusion.

#### 3.5.2 AUC: group level for both conditions

**Figure 6:**
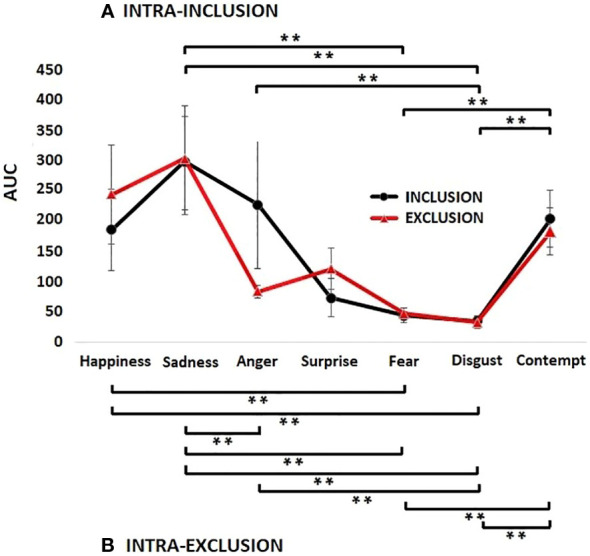
Mean values and standard errors of the total areas under the curve for each emotion during the condition of social inclusion (black lines) and social exclusion (red lines) while patients with Borderline Personality Disorder performed the Cyberball paradigm. Although surprise was nearly significant (*p<* 0.08) there were no significant differences between emotions across conditions. Significant differences between emotions of the same condition were represented with brackets for the condition of inclusion **(A)** and for the condition of exclusion **(B)** (**, *p<* 0.001). In both conditions, sadness, happiness, and contempt were the emotions with the highest values, and fear and disgust were the emotions with the lowest values. Overall, during exclusion, statistically greater significant differences between emotions were found.

In **Table 5**, the values of the AUC for each emotion and masked emotions are shown. Wilcoxon non-parametric tests were conducted to compare the condition of inclusion versus the one of exclusion. Happiness, anger, and surprise, as can be seen in **Figure 6**, were the emotions with the greatest changes, while sadness, fear, disgust, and contempt remained nearly constant across both experimental conditions; fear and disgust were the emotions with the lowest mean values reported. Happiness and surprise exhibited higher levels during exclusion; on the contrary, anger followed the opposite pattern, attaining higher levels during inclusion than during exclusion. However, no statistically significant differences were found between conditions, primarily due to large variations across subjects. Only surprise exhibited a tendency toward significance (*Z* = -1.72*, p<* 0.08).

**Table 5:** Means and standard errors (SE) of the calculated Areas Under the Curve (AUC) of each facial emotion expression for the conditions of social inclusion and exclusion and their comparisons and similar measures now for masked emotions are presented.

Area Under the Curve per Emotion				
***Emotion***	**Inclusion**	**Exclusion**	***Z* statistic**	***p*-value**
Happiness, mean (SE)	186.40 (68)	257.26 (310)	-1.601	0.109
Sadness, mean (SE)	300.23 (300)	320.21 (353)	-0.596	0.551
Anger, mean (SE)	228.74 (400)	89.59 (39)	-1.412	0.158
Surprise, mean (SE)	74.20 (120)	128.47 (128)	-1.726	0.084
Fear, mean (SE)	44.86 (46)	50.88 (37)	-1.224	0.221
Disgust, mean (SE)	35.52 (33)	35.37 (38)	-0.847	0.397
Contempt, mean (SE)	204.70 (176)	193.72 (148)	-0.094	0.925
***Masked emotions (emotion plus happiness)***	**Inclusion**	**Exclusion**	***Z* statistic**	***p*-value**
Sadness, mean (SE)	486.87 (136)	577.47 (154)	-1.852	0.064
Anger, mean (SE)	415 (118)	346.85 (78)	-0.094	0.925
Surprise, mean (SE)	260.84 (74)	385.73 (76)	-2.417	**0.016 **
Fear, mean (SE)	231.49 (75)	308.14 (85)	-1.852	0.064
Disgust, mean (SE)	222.15 (68)	292.63 (85)	-1.475	0.140
Contempt, mean (SE)	391.34 (79)	450.98 (99)	-1.099	0.272

In bold, the masked surprise was significantly different between the two experimental conditions (p = 0.016). Masked sadness and masked fear increased during exclusion and were nearly significant according to Wilcoxon tests.

Therefore, considering the polarized levels of each emotion, comparisons within the conditions were conducted between emotions. For inclusion, as observed in **Figure 6**, sadness, anger, and contempt were the emotions reporting the higher values, with mean AUC scores of 300.23 ± 306, 228.74 ± 400 and 204.70 ± 176, respectively. Happiness, surprise, fear, and disgust: 186 ± 255, 74.20 ± 120, 44.85 ± 46 and 35.51 ± 33, respectively, were the emotions that exhibited middle to lower mean values. Respecting the condition of inclusion, among the 21 possible combinations of emotions, significant differences were observed in the following pairs: happiness and fear (*Z* = -2.22, *p<* 0.02); happiness and disgust (*Z* = -2.22, *p<* 0.02); sadness and surprise (*Z* = -2.54, *p<* 0.01); anger and fear (*Z* = 2.41, *p<* 0.01); contempt and surprise (*Z* = -1.97, *p<* 0.04), sadness and fear (*Z* = -3.29, *p<* 0.001); sadness and disgust (*Z* = -3.29, *p<* 0.001); anger and disgust (*Z* = -3.29, *p<* 0.001); fear and contempt (*Z* = -2.73, *p<* 0.006); contempt and disgust (*Z* = -2.85, *p<* 0.004).

The emotions with significant differences for the condition of exclusion were as follows: fear and anger (*Z* = -2.04, *p<* 0.04); contempt and anger (*Z* = -2.29, *p<* 0.02), happiness and fear (*Z* = -2.66, *p<* 0.008); happiness and disgust (*Z* = -2.79, *p<* 0.005); sadness and anger (*Z* = -3.17, *p<* 0.002); sadness and fear (*Z* = -3.29, *p<* 0.001); sadness and disgust (*Z* = -3.23, *p<* 0.001); anger and disgust (*Z* = -3.23, *p<* 0.001); fear and contempt (*Z* = -3.23, *p<* 0.001); and contempt and disgust (*Z* = -3.23, *p<* 0.001).

In summary, respecting the condition of inclusion, five out of ten significant differences exhibited a *p<* 0.001, while in the condition of exclusion, eight out of the ten significant differences exhibited a *p<* 0.001. Therefore, considering the emotions with no segmentation, a noticeable contrast between emotions was found during the condition of exclusion compared to the condition of inclusion. During the condition of exclusion, the higher means of the AUC were sadness and happiness with 320.20 ± 353 and 257.26 ± 310, respectively, while contempt, surprise, anger, fear, and disgust, with 193.72 ± 148, 128.46 ± 128, 89.58 ± 39, 50.88 ± 37 and 35.37 ± 38, respectively, were the four emotions with the lower values. The black square brackets in **Figure 6A** represent the significant differences in emotions during inclusion. Analogously for the condition of exclusion, in **Figure 6B**, the square brackets below represent the significant differences.

#### 3.5.3 Summed values of the AUC: masking of emotions

The summed AUC of happiness and surprise yielded the only statistically significant result (*Z* = −2.41, *p<* 0.01), indicating an increase for exclusion versus inclusion. The mean AUC was 260.84 ± 227 for inclusion and 385.73 ± 285 for exclusion (**Table 5**). The individual values ranged from 6.72 to 1003.53 during the condition of inclusion and from 7.82 to 1086.46 during the condition of exclusion.

#### 3.5.4 Differences between the AUC of segments at group level

**Figure 7:**
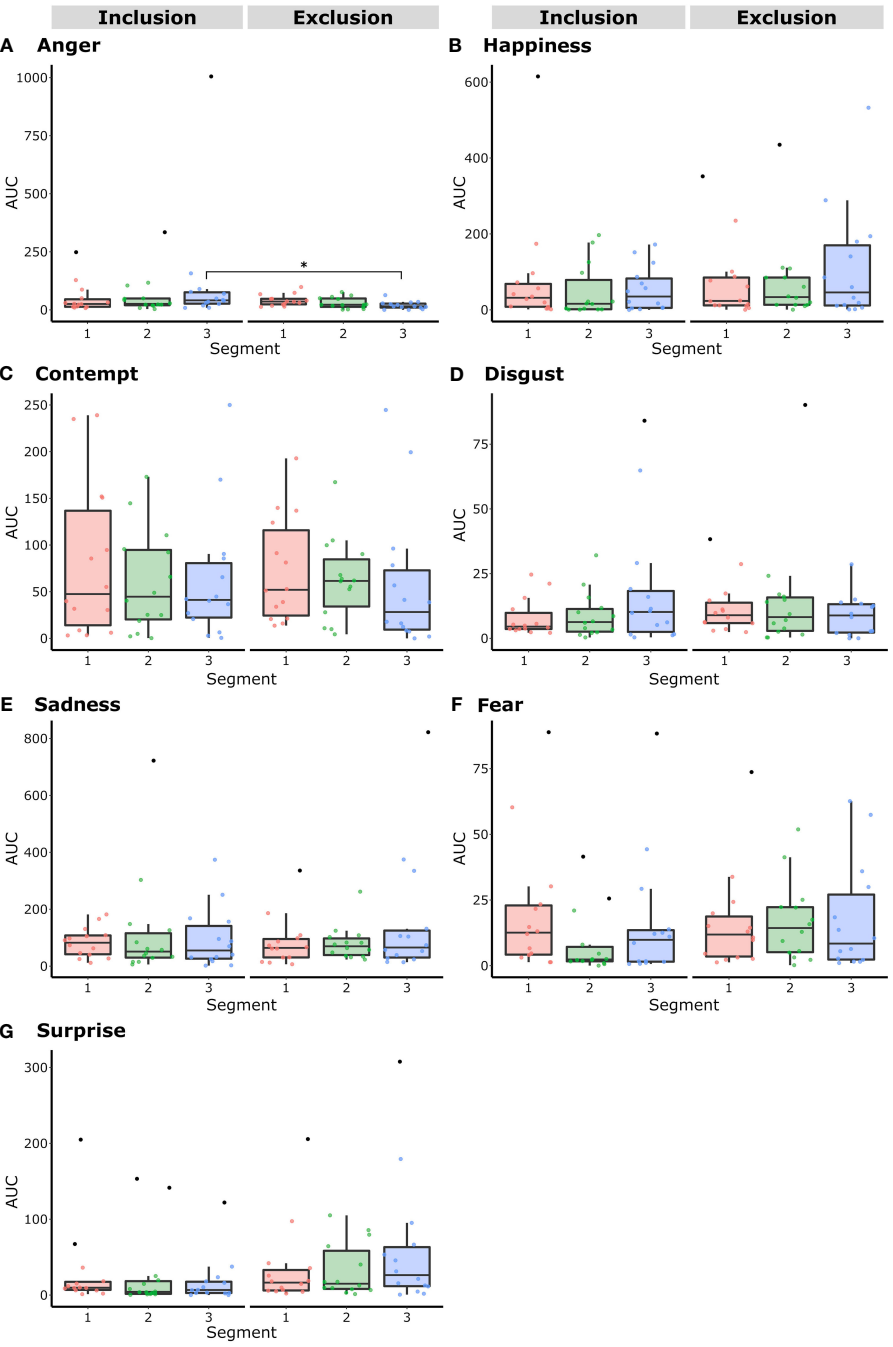
The Area Under the Curve (AUC) of the primary emotions **(A)** Anger, **(B)** Happiness, **(C)** Contempt, **(D)** Disgust, **(E)** Sadness, **(F)** Fear, and **(G)** Surprise in Borderline Personality Disorder expressions during the conditions of inclusion or exclusion in a Cyberball paradigm session, divided into time segments (1 to 3), were analyzed using box plots with outliers represented by black dots. Two-way repeated measures ANOVAs were performed to assess differences between conditions per segment. Anger demonstrated a significant interaction between the third segments of the conditions, being higher during inclusion than during exclusion (*p<* 0.01).

The analyses revealed surprise was statistically significant between conditions, and fear was near-significance, see **Table 6**. Notably, anger showed a significant interaction: it increased during inclusion and decreased for exclusion across segments (**Figure 7**). However, the power of each test, considering outliers, was low due to the small sample size: anger (0.18), disgust (0.05), happiness (0.10), sadness (0.05), fear (0.28), and surprise (0.08).

**Table 6:** Analysis of the area under the curve of each emotion by segments: Details of the two-way Repeated-Measures ANOVA for each analyzed variable, including outliers, are presented.

Variable	Effect	DFn	DFd	F	*p*	*η*2	*p* adjusted
**Anger**	**Condition**	1	13	2.826	0.117	0.077	0.580
	**Segm**	2	26	1.113	0.329	0.019	0.686
	**Condition:** **Segm**	2	26	6.975	*0.004*	0.084	0.078
**Contempt**	**Condition**	1	13	0.003	0.955	2.30E-05	0.975
	**Segm**	2	26	1.240	0.306	0.018	0.686
	**Condition: Segm**	2	26	0.401	0.674	0.004	0.821
**Disgust**	**Condition**	1	13	0.018	0.895	3.28E-04	0.975
	**Segm**	2	26	0.153	0.859	0.002	0.975
	**Condition:** **Segm**	2	26	2.039	0.150	0.030	0.580
**Happiness**	**Condition**	1	13	2.802	0.118	0.025	0.580
	**Segm**	2	26	0.783	0.468	0.008	0.686
	**Condition:** **Segm**	2	26	1.712	0.200	0.012	0.580
**Sadness**	**Condition**	1	13	0.541	0.475	0.005	0.686
	**Segm**	2	26	0.025	0.975	2.21E-04	0.975
	**Condition:** **Segm**	2	26	0.903	0.418	0.018	0.686
**Fear**	**Condition**	1	13	3.341	0.091	0.024	0.580
	**Segm**	2	26	1.663	0.209	0.031	0.580
	**Condition:** **Segm**	2	26	2.118	0.141	0.032	0.580
**Surprise**	**Condition**	1	13	6.398	*0.025*	0.078	0.263
	**Segm**	2	26	0.964	0.394	0.006	0.686
	**Condition:** **Segm**	2	26	1.591	0.223	0.020	0.580

This includes degrees of freedom in the numerator (DFn), degrees of freedom in the denominator (DFd), F critical value (F), and partial eta-squared (η^2^). p-values were adjusted using the Benjamini-Hochberg method. Italicized text indicates nearly significant results. Data are expressed as the mean ± s.e.m. (standard error of the mean) unless otherwise specified. Statistical differences were considered significant at p ≤ 0.05.

#### 3.5.5 Correlations between AUC

There were few significant correlation between AUC. Specifically, the AUC of happiness during exclusion was negatively correlated with the AUC of anger also during that condition (*r* = −0.56, *p<* 0.03). Similarly, the AUC of happiness was correlated between conditions (*r* = 0.65, *p<* 0.01); happiness during inclusion was positively correlated with disgust during exclusion (*r* = 0.55, *p<* 0.04). Fear during inclusion was positively correlated with happiness during exclusion (*r* = 0.58, *p<* 0.02). The highest positive correlations, however, were observed between fear across conditions (*r* = 0.70, *p<* 0.005) and contempt also during both conditions (*r* = 0.72, *p<* 0.003). Finally, sadness during inclusion and contempt during exclusion were negatively correlated (*r* = −0.53, *p<* 0.04).

#### 3.5.6 Correlations between AUC and clinimetric scales

**Figure 8:**
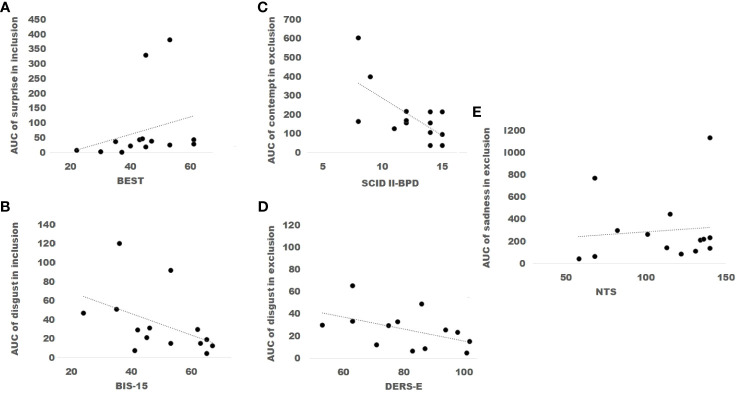
The scores of the Borderline Evolution of Severity Over Time (BEST) were positively correlated with the values of the Area under the curve (AUC) of surprise during the condition of inclusion **(A)**. In contrast, scores of the Barratt Impulsiveness Scale (BIS-15) were inversely correlated with the values of the AUC of disgust during inclusion **(B)**. Scores of the Structured Clinical Interview for DSM-IV (SCID II- BPD) were inversely correlated with the values of the AUC of contempt during exclusion **(C)**. Additionally, scores of the Difficulties in Emotion Regulation Scale, Spanish version, (DERS-E) were inversely correlated with the values of the AUC of disgust in the condition of exclusion **(D)**. Finally, scores of the Need Threat Scale (NTS) were positively correlated with the values of the AUC of sadness in exclusion **(E)**.

In **Table 7**, significant correlations are outlined. There was a significant and positive correlation between the SCID-II and surprise during inclusion (*r* = 0.54*, p<* 0.05). In contrast, a significant negative correlation was observed between SCID-II and contempt during exclusion (*r* = −0.53, *p<* 0.05). See **Figure 8**. Additionally, consistent with the aforementioned result, the BIS-15 and the AUC of disgust during both conditions were negatively correlated, with statistical significance reported only during the condition of inclusion (*r* = −0.57*, p<* 0.03). Moreover, as demonstrated in the respective table, specifically, the AUC of disgust during the condition of exclusion and DERS-E exhibited a significant negative correlation (*r* = −0.56, *p<* 0.05). Furthermore, the NTS was almost positively correlated with sadness during exclusion.

**Table 7:** Spearman’s correlations with Bonferroni corrections between the scores of the Structured Clinical Interview for DSM-IV (SCID-II), Barratt Impulsiveness Scale (BIS-15), Borderline Evaluation of Severity Over Time (BEST), the Need Threat Scale (NTS), and the values of areas under the curve per emotion are presented.

SCID-II						
	Inclusion			Exclusion		
	Spearman’s *r*	*p*	*p* adjusted	Spearman’s *r*	*p*	*p* adjusted
**Happiness**	-0.015	0.957	1	-0.071	0.807	1
**Sadness**	0.379	0.180	1	0.042	0.884	1
**Anger**	-0.321	0.263	1	-0.244	0.399	1
**Surprise**	0.541	*0.045*	0.630	0.186	0.523	1
**Fear**	0.312	0.277	1	0.330	0.249	1
**Disgust**	-0.352	0.216	1	-0.352	0.216	1
**Contempt**	-0.060	0.836	1	-0.538	*0.046*	0.644
BIS-15						
	Inclusion			Exclusion		
	Spearman’s *r*	*p*	*p* adjusted	Spearman’s *r*	*p*	*p* adjusted
**Happiness**	-0.118	0.685	1	-0.160	0.582	1
**Sadness**	-0.207	0.477	1	-0.140	0.630	1
**Anger**	-0.019	0.946	1	0.077	0.793	1
**Surprise**	-0.061	0.834	1	0.367	0.195	1
**Fear**	-0.019	0.946	1	0.279	0.332	1
**Disgust**	-0.579	*0.029*	0.406	-0.462	0.095	1
**Contempt**	0.081	0.781	1	-0.167	0.567	1
BEST						
	Inclusion			Exclusion		
	Spearman’s *r*	*p*	*p* adjusted	Spearman’s *r*	*p*	*p* adjusted
**Happiness**	0.185	0.526	1	0.165	0.572	1
**Sadness**	0.092	0.752	1	0.008	0.976	1
**Anger**	0.059	0.839	1	-0.355	0.212	1
**Surprise**	0.513	0.060	0.840	0.205	0.481	1
**Fear**	0.286	0.320	1	0.187	0.521	1
**Disgust**	-0.251	0.385	1	-0.229	0.430	1
**Contempt**	-0.222	0.444	1	-0.357	0.209	1
DERS-E						
	Inclusion			Exclusion		
	Spearman’s *r*	*p*	*p* adjusted	Spearman’s *r*	*p*	*p* adjusted
**Happiness**	-0.464	0.109	1	-0.426	0.146	1
**Sadness**	0.261	0.388	1	0.195	0.522	1
**Anger**	-0.112	0.713	1	0.167	0.583	1
**Surprise**	0.101	0.740	1	0.099	0.747	1
**Fear**	0.063	0.837	1	0.027	0.928	1
**Disgust**	-0.327	0.274	1	-0.561	*0.045*	0.630
**Contempt**	-0.184	0.546	1	-0.453	0.119	1
NTS						
	Inclusion			Exclusion		
	Spearman’s *r*	*p*	*p* adjusted	Spearman’s *r*	*p*	*p* adjusted
**Happiness**	-0.139	0.635	1	-0.008	0.976	1
**Sadness**	0.207	0.476	1	0.528	0.052	0.728
**Anger**	-0.077	0.792	1	0.097	0.740	1
**Surprise**	0.198	0.495	1	0.271	0.347	1
**Fear**	0.457	0.100	1	0.302	0.292	1
**Disgust**	0.035	0.904	1	0.015	0.958	1
**Contempt**	-0.103	0.723	1	-0.167	0.565	1

## 4 Discussion

The present research provided a detailed follow-up of single and mixed facial emotional expressions under two levels of analysis. This is the first study to present the automatized analysis of facial expression of patients with BPD during the experimental conditions of the Cyberball paradigm.

The only report on a longitudinal evaluation of emotions has been conducted by Williams (4), who utilized a “feelings dial” to facilitate the measurement of emotions on a Likert scale ranging from happy to sad. This study employed a second-by-second analysis of decision-making processes and found that, in individuals without psychiatric disorders, mood began to decline after 20 seconds without the ball.

To our knowledge, no other study has attempted to describe in detail the distribution of emotions and the configuration of blended or masked emotions involved in the reflexive stage, as mentioned by Williams (4), not only after the tenth ball throw (3) but throughout the entire game.

Previous studies have highlighted an apparent contradiction as one of the features of the disorder. In the present study, anger exhibited a significant interaction with each condition and segment, increasing during inclusion while decreasing during exclusion. Therefore, this specific emotion, documented as challenging to manage according to the literature on people with BPD (23), was observed during the condition of inclusion. The interaction of anger occurred during the third segment of each condition, even though patients felt more ostracized in the condition of exclusion compared to the condition of inclusion (NTS).

Social exclusion induced by the Cyberball paradigm has been consistently shown to elicit intense negative emotional reactions, such as ostracism, in individuals without psychiatric disorders (4, 24, 25). According to self-reports, patients with BPD are particularly susceptible to exclusion (3, 7, 26). However, during the Cyberball paradigm, which might shed light on the specific processing of emotional stimuli in BPD, they tend to underestimate their participation percentage in the game when they are included, as indicated by the NTS (3, 7). Recent research on the recognition of faces, considering effortful control, found that students with high BPD features and low control tended to accurately detect and identify subtle negative emotions. This supports the “empathy paradox” hypothesis. However, they failed to detect most neutral faces, labeling them as negative, which favors the hypothesis of a negativity bias (27).

This subjective bias among patients with BPD has also been corroborated by physiological responses observed during the Cyberball game. Lower levels of Respiratory Sinus Arrhythmia (RSA) were documented during the inclusion condition compared to an eyes-open resting state, while RSA levels remained consistently low during the exclusion condition (7). In such study, patients exhibited lower baseline RSA levels than both controls and patients with MDD prior to the start of both conditions. These findings were interpreted in light of the challenges individuals with BPD encounter when engaging in social interactions, stemming from an initial heightened activation response. Consistent with the hypothesis of the emotional modulation paradox in patients with BPD, reports of autonomic nervous system activations associated with fight-or-flight responses (i.e., increased heart rate) have been observed during both the Cyberball paradigm and interviews, rather than a predominant involvement of the ventral vagal parasympathetic branch, which is linked to the social engagement system (28).

The AUC of surprise when the segmentation was carried out and the masked emotions of surprise plus happiness increased during social exclusion. During this condition, individuals with BPD may appear to anticipate ostracism. Therefore, the expectation of experiencing greater surprise during this condition might seem counterintuitive. Nonetheless, the emotion of surprise was significant between conditions and also when it was masked. These reactions could be explained by some of the assumptions made by (7). Patients with BPD perceive inclusion as a challenging and threatening situation. As a result, the “vagal brake” is withdrawn, and anger is triggered, particularly during the third segment of inclusion.

Fight, flight, and freeze responses may be present, while social responses are inhibited. As the exclusion condition begins, patients’ emotions apparently remain unchanged until the participant suddenly stops receiving the ball. Surprise and happiness may serve as markers of the intention for social engagement, presumably accompanied by increases in RSA. Conversely, anger nearly dissipates.

Traditional literature indicates that the effects of ostracism in individuals without psychiatric disorders are akin to pain (4). During the Cyberball paradigm, control subjects exhibited higher levels of happiness (both felt and unfelt) compared to patients with BPD (3), although the differences between conditions were not significant.

It is noteworthy that Staebler et al. (3) found a higher prevalence of blended emotions in patients with BPD compared to healthy controls, particularly during the condition of exclusion (BPD group: 74% vs. control group: 18%). It is important to note that Staebler et al. (3) evaluated emotions both separately and jointly, allowing for the identification of unique emotions as well as mixed emotions (defined as “two emotional expressions displayed at the same time”). In contrast, in our study, emotions were detected and reported continuously throughout the game. In this sense, the different methodologies, the single but longitudinally evaluated group and the higher level of ostracism observed in the current study, compared to that of Staebler et al. (3), may be responsible for the discrepancies of the different percentages of blended emotions between conditions.

In the present study, during the condition of exclusion, anger was found to be negatively correlated with happiness.

Contempt and disgust were present in both conditions in a similar intensity, exhibiting disgust a negative correlation with impulsivity during inclusion and likewise with BPD symptomatology and difficulties in emotion regulation, during the condition of exclusion. Culicetto et al. (29) propose disgust as a transdiagnostic index of mental illness across various pathologies. According to their review, in BPD, poor recognition of others’ disgust is associated with increased activation in the insula and posterior cingulate cortex.

Schienle et al. (30) found that women diagnosed with BPD perceive facial expressions of disgust in a higher rate than their healthy counterparts, but solely in instances where the individual exhibiting this emotion was male. Additionally, the spectrum of self-disgust, trait disgust, and disgust recognition were positively associated with disorder severity. However, although the present results seem to contradict these findings, it must be recalled that in the present study the inverse correlation between expressed disgust was found under the condition of inclusion and previously measured impulsivity; furthermore, the negative associations between disgust and dysregulation and contempt and severity happened especially when social exclusion was being undertaken. Anger had disappeared and the patients seemed to be overwhelmed by feelings of sadness. These results seem to signify severe symptomatology is associated with a decrease in the expression of disgust during inclusion. Likewise, this relationship remains unchanged during ostracism, and the patient with BPD behaves in a similar way when expressing contempt.

In patients with BPD, Kot et al. (31) found that self-disgust is highly associated with alexithymia, emotion regulation, and comorbid psychopathology but with a lower degree of disgust sensitivity. In these subjects, Unoka et al. (32) found a higher attribution of disgust and surprise than a control group when the task consisted in recognizing Ekman faces.

Given that most of the patients in the present research underwent psychoeducational therapy, and a few received various modules including tolerance distress and emotional regulation, i.e., DBT, a greater expression of emotions than patients without treatment could be expected, which might be related to lower disorder severity. In this sense, if emotional dysregulation consists in alterations in the identification of emotions, the nonacceptance of emotional responses, in difficulties of engaging in goal-directed, in the lack of emotional awareness, and lack of emotional clarity, then, especially disgust and contempt may be attenuated when all these features are present in a greater proportion.

The study by (33) stated that patients with BPD were more prone to rate disgust in questionnaires when viewing images of the International Affective System than control subjects. A lower activation of the left amygdala and an increased activation of the dorsolateral prefrontal cortex and the ventral striatum were found. It could be assumed that aberrant processing of emotions among frontal and limbic regions may underlie not only emotional processing when rating images but also its expression during a relatively socially complex task such as the Cyberball paradigm.

Moreover, our findings suggest the presence of additional interactions between the scales and emotions of patients with BPD. For instance, the correlation between surprise during the condition of inclusion and the score of the BEST may indicate that greater surprise when being equally included is associated with increased severity. As stated before, the inverse correlation between disgust during the condition of inclusion and the score of the BIS-15 might indicate that the expression of disgust is attenuated when impulsivity is presented, but in the case of surprise, there is a straightforward association.

In previous studies, the observation of happiness in healthy controls seems to be a positive indicator rather than a negative one. In preliminary studies (34, 35), a higher level of happiness was found in both conditions in patients with BPD with medium indexes of mistrust. Future studies should explore whether individuals with BPD who display higher levels of happiness are more likely to experience faster improvements in therapy compared to those with explicitly higher levels of social mistrust.

Additionally, Staebler et al. (3) observed more blending of emotions in exclusion than in inclusion across the four groups. However, Staebler et al. (3) did not report the precise percentage of masking, such as happiness mixed with a negative emotion, explicitly. In the present study, precise percentages are reported, along with differences between conditions.

Specifically, in the pattern analysis with a 15-second resolution, happiness increased in the last segment of the game. However, in the AUC analysis with nearly 45 seconds, this increase was statistically significant but disappeared with the *p*-value correction. In this context, happiness could potentially serve as a protective mechanism against feelings of failure and abandonment, especially in certain patients. The expression of happiness during inclusion, being positively correlated with more disgust during exclusion, could be an indicator of proneness toward social involvement and a marker of lower difficulty in emotion regulation. Drawing on insights from Gunderson and Lyons-Ruth (36), further research could investigate whether these variables indeed serve as markers that inform therapeutic decisions. 

Moreover, sadness was the most commonly expressed emotion measured during both conditions. In our study, patients with BPD exhibited a high comorbidity of MDD and were medicated, suggesting that the elevated levels of sadness observed may reflect this comorbidity. Interestingly, in one study, researchers found reduced facial reactivity in BPD patients, as measured by electromyography, when recognizing facial expressions, despite their explicit reports of stronger subjective responses to negative emotions (37). Additionally, (3) also observed more negative emotions expressed in the groups of patients with BPD compared to healthy controls, although no differences were observed in the overall measurements of emotions between experimental conditions.

The discovery of the relatively high frequency of sadness throughout the entire experimental conditions, and the positive correlation between the intensity of sadness and feeling more ostracized during exclusion along with the observation that some profiles displayed anger during inclusion but decreased in the third segment during social exclusion, is a novel finding not previously reported. Similarly, the correlation observed between sadness during the condition of exclusion and the NTS score might signify patients seeking support during moments of exclusion. Some of these interactions may appear intuitively expected and thus warrant thorough investigation in future research.

Several studies have confirmed that even after social inclusion, patients with BPD report feelings of exclusion (3, 7). In our study, patients expressed more feelings of being excluded during the exclusion phase than during inclusion (NTS). This could be attributed to the fact that they were more severely ostracized than in previous studies, where the percentage of exclusion was lower.

In mental disorders such as post-traumatic stress disorder following an earthquake (38) and eating disorders (39), computerized analyses of facial expression have been useful for evaluating reactivity. Statistical analyses of outputs from the FaceReader system vary widely across the literature. Different approaches, such as linear regression (38), normalization of data with arcsine transformation followed by mixed-effects linear modeling, and correlations between scores (39), or calculation of means and standard deviations for each emotion (40), have been employed. In contrast, this study utilized behavioral data as outputs, employing various alternative processing methods such as blending and masking of emotions at both individual and group levels to compare conditions and timings. While pattern analysis offers a more detailed and illustrative follow-up of patients based on percentages, AUC analysis considers significant increases, as shown in the experimental protocol, see **Figure 1**. In this sense, in the best-case scenario, the pattern, and AUC analyses derived from the FaceReader software could be used hereafter to extract the principal features of the automatic facial expression during the Cyberball paradigm. Noteworthy, the implementation of shuffled analysis has shed light upon the differences between actual natural patterns of facial expressions and random ones (41).

The emotions expressed by patients with BPD, as revealed through interviews, have been associated with treatment outcomes (42–44). By employing more precise temporal tracking of the expressions of patients with BPD during both conditions, one of the aims of the proposed analyses was to gather and integrate various data acquired from the patient, including interviews, self-reports, and computer-based techniques, to identify more accurate diagnoses and consequently, predictors of individualized therapies to determine probable prognosis (45).

## 5 Limitations

Due to the small sample size and/or the heterogeneity of BPD pathology related to a well-known concept in BPD literature or the degree of rejection sensitivity, see Gunderson and Lyons-Ruth (36, 46), in the group analysis, there were no significant differences between conditions when corrections were made. The virtual players in this study are simulated entities, not actual individuals. Additionally, the sample exclusively consisted of women, which may limit the generalizability of the findings. Furthermore, comparisons with control subjects and other psychiatric disorders were not conducted. Neutral faces were also excluded from the analyses. It is worth noting that this research commenced prior to the onset of the COVID-19 pandemic, and therefore, the mandate for wearing masks in enclosed spaces in Mexico during subsequent years limited the scope of this study.

## Data availability statement

The raw data supporting the conclusions of this article will be made available by the authors, without undue reservation.

## Ethics statement

The studies involving humans were approved by Comité de Ética en Investigación del INPRFM. The studies were conducted in accordance with the local legislation and institutional requirements. The participants provided their written informed consent to participate in this study.

## Author contributions

IA-d-M: Conceptualization, Data curation, Funding acquisition, Investigation, Methodology, Project administration, Resources, Software, Supervision, Validation, Writing – review & editing. AR-S: Conceptualization, Data curation, Investigation, Methodology, Project administration, Resources, Software, Writing – original draft. AR-L: Formal analysis, Methodology, Resources, Visualization, Writing – original draft, Writing – review & editing. M-LE-D: Formal analysis, Methodology, Resources, Visualization, Writing – review & editing. EV-M: Formal analysis, Resources, Visualization, Writing – review & editing. AR-D: Conceptualization, Methodology, Software, Supervision, Writing – review & editing. JM-D: Conceptualization, Writing – review & editing. IV-M: Methodology, Software, Writing – review & editing. ER-T: Methodology, Conceptualization, Investigation, Visualization, Software, Writing – review & editing.
